# Depressive symptoms and mortality – effect variation by body mass index: a prospective study in a primary care population

**DOI:** 10.1038/s41366-023-01296-3

**Published:** 2023-03-28

**Authors:** Ansa Talvikki Rantanen, Hannu Kautiainen, Päivi Elina Korhonen

**Affiliations:** 1 Department of General Practice, University of Turku and Turku University Hospital, 20520 Turku, Finland; 2 Folkhälsan Research Center, Helsinki, Finland; 3 Institute of Public Health and Clinical Nutrition, University of Eastern Finland, Kuopio, Finland

**Keywords:** Risk factors, Obesity

## Abstract

**Background/Objective:**

Pre-existing diseases have been found to affect the relationship between body mass index (BMI) and mortality. However, psychiatric disorders common in general population have not been previously addressed. The aim of this study was to assess the relationship of depressive symptoms and BMI with all-cause mortality.

**Methods:**

A prospective cohort study in Finnish primary care setting was conducted. A population survey identified 3072 middle-aged subjects who had elevated cardiovascular risk. Subjects who attended clinical examination and completed Beck’s Depression Inventory (BDI) (*n* = 2509) were included in this analysis. Effect of depressive symptoms and BMI on all-cause mortality after 14 years follow-up was estimated in models adjusted for age, sex, education years, current smoking, alcohol use, physical activity, total cholesterol, systolic blood pressure, and glucose disorders.

**Results:**

When subjects with and without increased depressive symptoms were compared, the fully adjusted hazard ratios (HR) for all-cause mortality in the BMI categories (<25.0, 25.0–29.9, 30.0–34.9, ≥35.0 kg/m^2^) were 3.26 (95% CI 1.83 to 5.82), 1.31 (95% CI 0.83 to 2.06), 1.27 (95% CI 0.76 to 2.11), and 1.25 (95% CI 0.63 to 2.48), respectively. The lowest risk of death was among non-depressive subjects who had BMI < 25.0 kg/m^2^.

**Conclusions:**

Effect of increased depressive symptoms on all-cause mortality risk seems to vary with BMI. Elevated mortality risk is especially apparent among depressive subjects with normal weight. Among individuals with overweight and obesity, increased depressive symptoms seem not to further increase all-cause mortality.

## Introduction

General adiposity, measured by body mass index (BMI), is associated with all-cause mortality [1]. There seems to be a J-shaped dose-response with increasing BMI, and lowest mortality risk is associated with normal weight (BMI 20.0–24.9 kg/m^2^) [2, 3]. The shape and magnitude of this relationship varies depending on whether smoking and pre-existing diseases have been taken into account [4, 5]. However, psychiatric disorders common in general population have not been addressed in these studies.

Depression or increased depressive symptoms have constantly been associated with increased risk of overweight and obesity, and vice versa [6]. This relationship can be explained by various mechanisms including complex biological pathways [7], lifestyle [8, 9], and psychological aspects [10]. Many of those mechanisms also contribute to mortality risk associated with both conditions. Not only depression but also milder depressive symptoms seem to increase all-cause mortality [11].

We have previously found that among an apparently healthy middle-aged population from primary care, especially non-melancholic depressive symptoms are associated with all-cause mortality [12]. In this study, our aim was to assess if the relationship between depressive symptoms and mortality is modified by BMI. We hypothesized that BMI is an effect modifier in the relationship between depressive symptoms and mortality.

## Methods

### Study population

Formulation of the study population is illustrated in Supplementary fig. 1. The study sample was drawn from the Harjavalta Risk Monitoring for Cardiovascular Disease (Harmonica) Project. It is a population survey conducted in the Finnish towns of Harjavalta and Kokemäki from autumn 2005 to autumn 2007. An invitation letter alongside with a CVD risk factor survey, a type 2 diabetes (T2D) risk assessment form (FINDRISC, Finnish Diabetes Risk Score) [13], and a tape for waist circumference measurement was mailed to all non-institutionalized 45–70-year-old inhabitants (*n* = 6103). Response rate was 74% (*n* = 4450). A subject was considered to be at CVD risk if she/he was having at least one of the assessed risk factors: waist circumference ≥80 cm in women and ≥94 cm in men (only in Harjavalta), use of antihypertensive medication, latest blood pressure (BP) measurement ≥140/90 mmHg, family history of ischemic heart disease, myocardial infarction, or stroke, history of gestational diabetes or hypertension, or FINDRISC-score ≥12 in Harjavalta or ≥15 in Kokemäki. Because of the primary preventive focus, subjects with previously established CVD, chronic kidney disease, or T2D were excluded. CVD risk subjects (*n* = 3072) were invited for laboratory tests and an appointment with a study nurse. 2752 subjects participated. In this study, only subjects with completed Beck’s Depression Inventory (BDI) [14] and information on weight and height were included (*n* = 2509).

### Questionnaires and measurements

Self-administered questionnaires were filled in before the study visit. Depressive symptoms were measured by the BDI [14], and the definition of increased depressive symptoms was a BDI score ≥10 [15]. Other forms gathered information on education, current smoking (yes/no), alcohol consumption by Alcohol Use Disorders Identification Test (AUDIT) [16], and current frequency of leisure-time physical activity (LTPA) for at least 30 min at a time. LTPA was categorized as low, moderate, or high as follows: at most three, four to five, at least six times a week, respectively. Self-rated health (SRH) was assessed by the Short-Form Health Survey (SF-36), version 1.0 [17], whose first question “In general, how would you rate your health” assesses SRH from 1 = poor to 5 = excellent. Health-related quality of life (HRQoL) was assessed by the EQ-5D-3L comprising five dimensions of health: mobility, self-care, usual activities, pain/discomfort, and anxiety/depression [18]. Each dimension is assessed on a 3-point scale; level 1 (no problems), level 2 (some problems), and level 3 (extreme problems).

The study visit was performed by a trained nurse. Height and weight were measured with subjects in a standing position without shoes and outer garments, and BMI was calculated by dividing weight (kg) by height squared (m^2^). BMI was categorized as: normal weight BMI ≤ 25.0 kg/m^2^, overweight BMI 25.0–29.9 kg/m^2^, obesity BMI 30.0–34.9 kg/m^2^, and severe obesity BMI ≥ 35.0 kg/m^2^. In addition, waist circumference at the level midway between the lower rib margin and the iliac crest and BP were measured. A 2 h oral glucose tolerance test (OGTT) was performed, and fasting plasma lipids were determined.

### Primary preventive interventions

All subjects attending the study visit were given lifestyle counselling by the study nurse. A referral to a physician was made for high risk subjects (*n* = 1928) with hypertension, diabetes, impaired glucose tolerance, metabolic syndrome, obesity (BMI ≥ 30.0 kg/m^2^), or ≥5% ten-year risk for CVD death estimated by the Systematic Coronary Risk Evaluation (SCORE). [19] With such a risk for fatal CVD event, preventive medication for hypertension or dyslipidaemia, or low dose aspirin, was initiated. According to national guidelines at that time, antihypertensive medication was initiated if systolic BP was ≥160 mmHg, diastolic BP was ≥100 mmHg, or target organ damage was diagnosed and intensified if systolic BP was ≥140 mmHg or diastolic BP was ≥85 mmHg (≥80 mmHg in patients with diabetes).

### Mortality

Data on mortality was obtained from the Official Statistics of Finland provided by Statistics Finland. Causes of death were classified according to the International Statistical Classification of Diseases and Related Health Problems, 10th Revision (ICD-10). A subject’s follow-up time started at the time of the study visit (9/2005 to 3/2008) and ended on December 31^st^, 2019.

### Statistical analysis

The descriptive statistics are presented as means with standard deviation (SD) or counts with percentages. Statistical comparison of characteristics of the subjects was evaluated using generalized linear models with appropriate distribution and link function; main effects of BMI and depressive symptoms, and their interaction. The expected number of deaths was calculated on the basis of sex-, age- and calendar period- specific mortality rates in the Finnish population (data from the official statistics from Statistics Finland). The expected number was determined by multiplying the person- years of observation by the appropriate mortality rate in the general population according to categories of sex, 1- year age group and calendar period (subject-years method) [20]. The standardized mortality ratio (SMR) was calculated as the ratio of observed and expected number of deaths for all-cause mortality with 95 percent confidence intervals (95% CI), assuming a Poisson distribution. Kaplan-Meier method was used to estimate the cumulative mortality and was compared between groups with the log-rank test. Cox proportional hazards regression was used to estimate the adjusted hazard ratios (HR) and their 95% CIs. Models were adjusted for age, sex, education years, current smoking, AUDIT score, LTPA, total cholesterol, systolic blood pressure, and glucose disorders. A possible non-linear relationship between BMI and the probability of increased depressive symptoms and hazard of death was assessed by using the 4-knot restricted cubic spline Cox proportional hazards model. The knots were located at the 5th, 35th, 65th and 95th percentiles, based on Harrell’s recommended percentiles. [21] Stata 17.0 (StataCorp LP) statistical package was used for the analysis.

### Ethical approval

The ethics committee of Satakunta Hospital District reviewed and approved the study protocol and consent forms. All participants provided written informed consent for the project and subsequent research.

### Patient and public Involvement

Patients and the public were not involved in the planning, design, conduct, or reporting of this research.

## Results

### Relationship of increased depressive symptoms and BMI

**Fig. 1:**
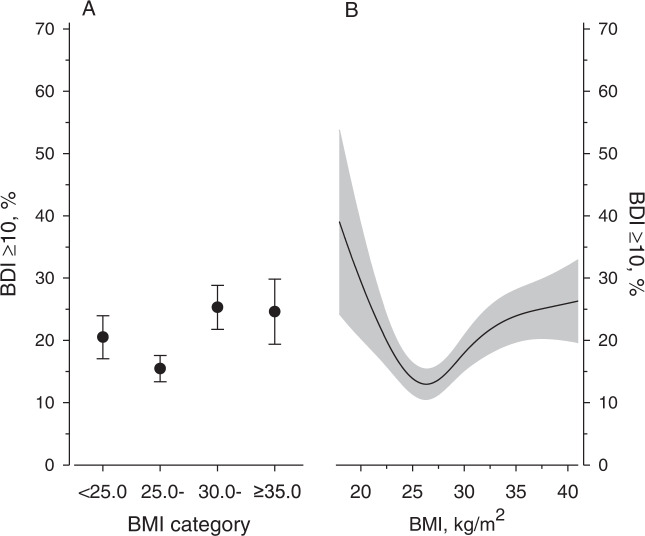
Estimated probability of increased depressive symptoms according to body mass index (BMI). **A** BMI as a categorized variable, **B** as a continuous variable. Increased depressive symptoms, Beck’s Depression Inventory (BDI) score ≥10. Adjusted for age, sex, education years, current smoking, Alcohol Use Disorders Identification Test score, leisure-time physical activity, total cholesterol, systolic blood pressure, and glucose disorders. Whiskers and grey area represent 95% confidence intervals.

The probability of increased depressive symptoms (BDI ≥ 10) according to categorized and in continuous BMI level was examined in models adjusted for age, sex, education years, current smoking, AUDIT score, LTPA, total cholesterol, systolic BP, and glucose disorders (Fig. 1A, B). BMI 25.0–29.9 kg/m^2^ was associated with the lowest proportion of increased depressive symptoms (*p*-value <0.001 for difference between categories). The curve relating continuous BMI and dichotomized BDI was U-shaped (*p* = 0.009, quadratic contrast).

### Characteristics of the study population

Baseline characteristics of the subjects according to categories of BMI and presence of increased depressive symptoms (BDI ≥ 10) (main effects and their interaction effects) are presented in Table 1. Subjects with higher BMI were more likely to be older, less educated and employed, and they had worse SRH and QoL. BMI was positively associated with plasma LDL-C, triglycerides, and glucose concentrations, prevalence of glucose disorders, and BP values. BMI was negatively associated with current smoking, LTPA, and HDL-C. Subjects with BDI ≥ 10 were more often females, older, less employed, used more alcohol, and performed less LTPA than non-depressive subjects. The proportion of women was especially high among those with BMI ≥ 35 kg/m^2^. Those with BDI ≥ 10 had worse SRH, and HRQoL. Depressive symptoms were also positively associated with plasma total cholesterol, triglyceride, and 2-hour glucose concentrations. The interaction between BMI and BDI was significant only for AUDIT score, level of LTPA, HRQoL, and diastolic BP. Distribution of BDI scores in different BMI categories is displayed in Supplementary fig. 2.

**Table 1:** Characteristics of the subjects according to categories of body mass index (BMI) and presence of depressive symptoms.

	Body Mass Index, kg/m^2^								*P*-value		
	<25.0		25.0–29.9		30.0–34.9		≥35.0		Main effect		Interaction
									BMI*	BDI	
	BDI < 10 *N* = 428	BDI ≥ 10 *N* = 107	BDI < 10 *N* = 984	BDI ≥ 10 *N* = 171	BDI < 10 *N* = 409	BDI ≥ 10 *N* = 145	BDI < 10 *N* = 192	BDI ≥ 10 *N* = 73			
Females, *n* (%)	280 (65)	70 (65)	458 (47)	103 (60)	215 (53)	85 (59)	122 (64)	59 (81)	0.31	<0.001	0.10
Age, years, mean (SD)	56 (7)	58 (7)	58 (7)	59 (7)	58 (7)	60 (6)	58 (6)	58 (7)	0.020	0.003	0.81
Education years, mean (SD)	10.8 (2.7)	10.6 (2.7)	10.4 (2.6)	10.4 (2.8)	10.4 (2.8)	10.0 (2.8)	10.1 (2.6)	10.5 (3.1)	0.021	0.70	0.48
Cohabiting, *n* (%)	329 (77)	82 (77)	802 (82)	130 (76)	311 (76)	106 (73)	149 (78)	54 (74)	0.39	0.17	0.78
Employment status, *n* (%)									0.006	<0.001	0.33
Employed	269 (63)	49 (46)	529 (54)	69 (40)	227 (56)	39 (27)	84 (44)	22 (30)			
Unemployed	22 (5)	8 (7)	52 (5)	13 (8)	24 (6)	14 (10)	17 (9)	8 (11)			
Retired	137 (32)	50 (47)	403 (41)	89 (52)	158 (39)	92 (63)	91 (47)	43 (59)			
Current smoking, *n* (%)	87 (20)	28 (26)	163 (17)	37 (22)	62 (15)	26 (18)	32 (17)	10 (14)	0.008	0.27	0.25
AUDIT score, mean (SD)	3.9 (4.2)	6.0 (6.3)	4.8 (4.7)	5.6 (5.8)	4.6 (4.9)	5.4 (5.9)	4.2 (4.3)	4.2 (5.0)	0.10	<0.001	0.017
Harmful alcohol use, *n* (%)	60 (14)	28 (26)	200 (20)	47 (27)	86 (21)	39 (27)	35 (18)	16 (22)	0.60	<0.001	0.16
Leisure-time physical activity, *n* (%)									<0.001	0.002	0.006
Low	55 (13)	14 (13)	131 (13)	29 (17)	86 (21)	43 (30)	58 (30)	36 (49)			
Moderate	211 (49)	52 (49)	511 (52)	83 (49)	209 (51)	70 (48)	99 (52)	31 (42)			
High	161 (38)	41 (38)	337 (34)	59 (35)	114 (28)	32 (22)	35 (18)	6 (8)			
Good self-rated health, *n* (%)	328 (77)	50 (47)	689 (70)	56 (33)	260 (64)	26 (18)	102 (53)	14 (19)	<0.001	<0.001	0.12
EQ-5D score, mean (SD)	0.887 (0.120)	0.785 (0.160)	0.871 (0.142)	0.695 (0.214)	0.838 (0.153)	0.671 (0.186)	0.810 (0.190)	0.622 (0.234)	<0.001	<0.001	0.003
Plasma lipids, mmol/l, mean (SD)											
Total cholesterol	5.33 (0.93)	5.35 (1.01)	5.39 (0.94)	5.60 (1.15)	5.32 (0.99)	5.49 (1.00)	5.36 (0.97)	5.40 (1.06)	0.88	0.038	0.81
LDL cholesterol	3.12 (0.83)	3.07 (0.82)	3.26 (0.86)	3.40 (0.98)	3.22 (0.90)	3.40 (0.98)	3.30 (0.90)	3.24 (0.96)	0.014	0.27	0.61
HDL cholesterol	1.74 (0.44)	1.76 (0.49)	1.56 (0.45)	1.58 (0.45)	1.42 (0.37)	1.43 (0.38)	1.35 (0.36)	1.37 (0.33)	<0.001	0.45	0.88
Triglycerides	1.12 (0.65)	1.15 (0.60)	1.33 (0.69)	1.47 (0.82)	1.54 (0.73)	1.70 (0.89)	1.66 (0.76)	1.82 (0.96)	<0.001	0.002	0.28
Fasting glucose, mmol/l, mean (SD)	5.32 (0.73)	5.56 (1.84)	5.55 (1.07)	5.45 (0.81)	5.73 (1.12)	5.77 (1.00)	6.18 (1.54)	6.28 (1.87)	<0.001	0.25	0.64
2 h glucose, mmol/l, mean (SD)	6.81 (1.66)	7.24 (2.28)	7.15 (2.01)	7.15 (2.18)	7.72 (2.22)	8.12 (2.85)	8.45 (2.90)	8.69 (3.07)	<0.001	0.029	0.98
Glucose tolerance, n (%)									<0.001	0.55	0.77
Normal	349 (82)	82 (77)	720 (73)	131 (77)	266 (65)	89 (61)	97 (51)	37 (51)			
Prediabetes	71 (17)	22 (21)	230 (23)	37 (22)	117 (29)	44 (30)	54 (28)	20 (27)			
Diabetes	8 (2)	3 (3)	34 (3)	3 (2)	26 (6)	12 (8)	41 (21)	16 (22)			
Blood pressure, mmHg, mean (SD)											
Systolic	137 (19)	138 (20)	140 (18)	140 (17)	142 (18)	140 (17)	146 (20)	141 (19)	<0.001	0.22	0.055
Diastolic	82 (10)	82 (10)	84 (9)	85 (10)	87 (10)	85 (10)	88 (11)	85 (10)	<0.001	0.10	0.010

*P-value for linearity. LTPA was tested on ordinal categorization. BDI < 10 = not increased depressive symptoms, BDI ≥ 10 = increased depressive symptoms. *BDI* Beck’s Depression Inventory, *AUDIT* Alcohol Use Disorders Identification Test, *HDL* high-density lipoprotein, *LDL* low-density lipoprotein.

### Mortality

In total, 32,216 person-years were followed up, and 289 deaths occurred. Number of person-years and deaths, crude, and standardized mortality ratio (SMR) according to BMI categories and depressive symptoms are displayed in Table 2. In subjects without increased depressive symptoms, SMR was 0.41 (95% CI 0.28 to 0.61) among those with BMI < 25 kg/m^2^ and rose linearly with increasing BMI (*p* = 0.002). In subjects with increased depressive symptoms and BMI < 25 kg/m^2^, SMR was 1.51 (95% CI 1.00 to 2.30), and no rise was noticed according to categories of BMI. (Table 2)

**Table 2:** Mortality data according to body mass index and depressive symptoms.

	Body Mass Index category, kg/m^2^			
	<25.0	25.0–29.9	30.0–34.9	≥35.0
Not increased depressive symptoms (BDI < 10)				
Person-years followed up	5669	12705	5239	2430
Number of deaths	25	112	44	28
Crude mortality over 14 years, % (95% CI)	6.0 (4.1 to 8.8)	11.9 (10.0 to 14.3)	11.5 (8.6 to 15.2)	14.1 (9.9 to 19.9)
Standardized mortality ratio (95% CI)	0.41 (0.28 to 0.61)	0.66 (0.55 to 0.79)	0.68 (0.50 to 0.91)	1.03 (0.71 to 1.49)
Increased depressive symptoms (BDI ≥ 10)				
Person-years followed up	1312	2147	1809	905
Number of deaths	22	23	23	12
Crude mortality over 14 years, % (95% CI)	21.5 (14.6 to 31.1)	13.6 (9.2 to 19.7)	18.3 (12.2 to 26.9)	16.4 (5.7 to 27.1)
Standardized mortality ratio (95% CI)	1.51 (1.00 to 2.30)	0.85 (0.57 to 1.28)	0.90 (0.59 to 1.35)	1.28 (0.73 to 2.26)

*BDI* Beck’s Depression Inventory, *CI* confidence interval. Cumulative mortality rate was estimated by Kaplan–Meier method. The standardized mortality ratio (SMR) was calculated as the ratio of observed and expected number of deaths for all-cause mortality. The expected number of deaths was calculated on the basis of sex-, age- and calendar-period-specific mortality rates in the Finnish population.

#### Cumulative all-cause mortality

**Fig. 2:**
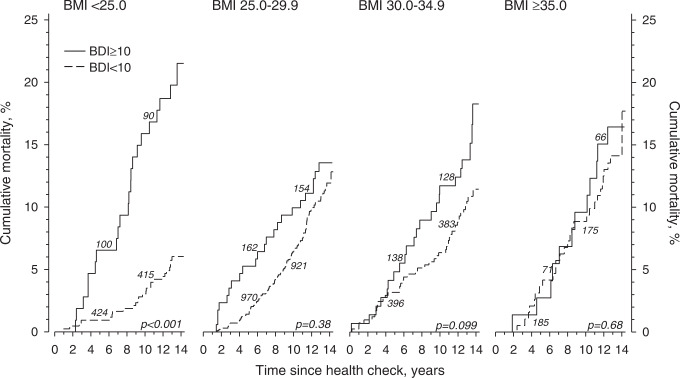
Crude cumulative mortality according to depressive symptoms in different body mass index (BMI) categories. In Italics the numbers of subjects at risk at 5 and 10 years. Beck´s Depression Inventory (BDI) score < 10, not increased depressive symptoms; BDI ≥ 10, increased depressive symptoms.

Unadjusted cumulative all-cause mortality according to depressive symptoms in the BMI categories is presented in Fig. 2. Only among subjects with BMI < 25.0 kg/m^2^, cumulative all-cause mortality was significantly higher in those with than without depressive symptoms.

#### Risk for all-cause mortality

**Fig. 3:**
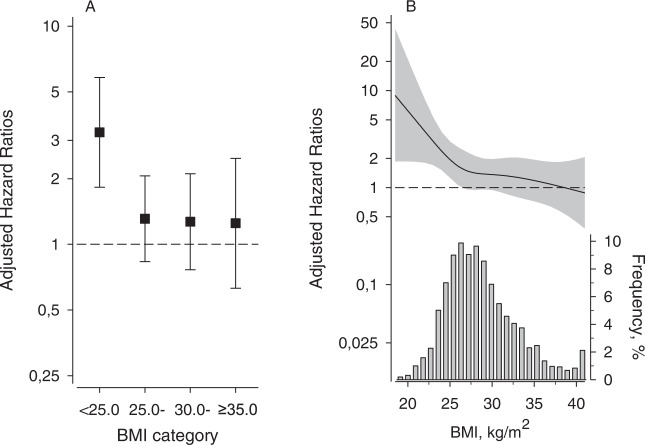
Adjusted hazard ratios for all-cause mortality between subjects with and without increased depressive symptoms according to body mass index (BMI). **A** BMI as a categorized variable, **B** as a continuous variable. Distribution of BMI is shown in (**B**). The continuous model (**B**) was derived from a 4-knots restricted cubic splines Cox regression model. Increased depressive symptoms, Beck’s Depression Inventory (BDI) score ≥10; not increased depressive symptoms, BDI <10. The models were adjusted for age, sex, education years, current smoking, Alcohol Use Disorders Identification Test score, leisure-time physical activity, total cholesterol, systolic blood pressure, and glucose disorders. Whiskers and grey area represent 95% confidence intervals.

When depressive and non-depressive subjects within a certain BMI category were compared, depressive symptoms increased fully adjusted risk for all-cause mortality only among subjects with normal weight (BMI < 25.0 kg/m^2^) [HR 3.26 (95% CI 1.83 to 5.82)] (Fig. 3, panel A). When BMI was handled as a continuous variable, adjusted HR for all-cause mortality decreased with increasing BMI (*p* = 0.015) (Fig. 3, panel B). The effect of depressive symptoms and BMI category on all-cause mortality is shown in Table 3 (*p* = 0.036 for interaction between BDI and BMI).

**Table 3:** Effect of depressive symptoms and body mass index category on all-cause mortality. The model adjusts for age, sex, education years, current smoking, Alcohol Use Disorders Identification Test score, leisure-time physical activity, total cholesterol, systolic blood pressure, and glucose disorders.

Body mass index category, kg/m^2^	Not increased depressive symptoms (BDI < 10) HR (95% CI)	Increased depressive symptoms (BDI ≥ 10) HR (95% CI)
<25.0	1.00 (Reference)^a^	3.26 (1.83 to 5.82)
25.0–29.9	1.55 (1.00 to 2.40)	2.04 (1.15 to 3.60)
30.0–34.9	1.57 (0.96 to 2.57)	1.99 (1.13 to 3.52)
≥35.0	2.09 (1.21 to 3.60)	2.61 (1.29 to 5.29)

^a^Denominator of hazard ratio (HR). *P* = 0.036 for interaction between BDI and BMI. *BDI* Beck´s Depression Inventory.

Causes of death according to depressive symptoms and BMI categories are presented in Supplementary table 1. Most prevalent causes of death were cancer (42.6%) and CVD (28.7%).

## Discussion

During 14 year-follow-up of 2509 CVD risk subjects from primary care, effect of depressive symptoms on all-cause mortality risk was found to vary with BMI. Increased depressive symptoms were associated with elevated mortality risk specifically among subjects with normal weight. Among subjects with overweight and obesity, depressive symptoms did not seem to further increase all-cause mortality.

Every one in five had increased depressive symptoms in this population-based sample of apparently healthy middle-aged subjects. Even after adjustment for several sociodemographic, lifestyle, and CVD risk factors, overweight (BMI 25.0–29.9 kg/m^2^) was associated with the lowest prevalence of depressive symptoms whereas more than one in four of the individuals with obesity (BMI ≥ 30.0 kg/m^2^) were depressive. Also previously the association of depressive symptoms and BMI has been proposed to be U-shaped rather than linear [22, 23]. Similar to our findings, the nadir of the curve has been found to be within the overweight range [22, 23]. For example, among over 43 000 adults from general population in the Netherlands, categorial and continuous self-reported BMI showed an U-curved association with self-reported depressive symptoms when adjusted for socio-demographic variables [22]. As the investigators pointed out this is plausible, as depression might be associated with increased or decreased appetite, and thus, weight gain or loss. Also among over 7000 Korean middle-aged and older adults, the lowest levels of depressive symptoms were found among subjects with overweight [23]. On the other hand, a recent meta-analysis suggests that depression or depressive symptoms are more prevalent among subjects with overweight and obesity compared to those with normal weight [24], whereas in prospective studies, only obesity has been found to be bidirectionally associated with depressive symptoms [6].

Although several studies on the association of mortality with BMI and mortality with depressive symptoms have been conducted, we are not aware that the association of BMI, depressive symptoms, and mortality would have previously been studied simultaneously. This is somewhat surprising given it is well-known that both these conditions are associated with mortality [1, 11], and that they are interconnected [6]. Previous research has found a J-or U-shaped association of BMI with all-cause mortality, those with normal weight at lowest risk [2–4], and suggested 5 to 39% increased risk for mortality per 5 kg/m^2^ units higher BMI beyond normal weight [1, 3, 4]. For example, a meta-analysis from 239 prospective studies controlled for reverse causality of pre-existing disease (e.g. CVD, cancer, or respiratory diseases) and ever-smoking suggested rather log-linear 39% increase in mortality risk with every 5 unit increase in BMI in European studies [1]. Similarly, one of the most recent meta-analyses considering mortality and depression suggests relative risk of 1.71 for mortality with depression [25]. Among Finnish middle-aged population, Markkula et al. have found that mild or moderate (BDI 10–18) and severe (BDI ≥ 19) depressive symptomatology increase all-cause mortality risk by 53 and 77 % accordingly, when adjusted for socio-economic factors, health status, and smoking [26].

In our study, depressive symptoms were found to affect all-cause mortality risk varying with BMI. Compared to non-depressive subjects with normal weight, mortality risk was elevated among depressive subjects in all BMI categories. This risk increase was especially apparent among subjects with BMI < 25 kg/m^2^, and higher than previous studies suggest. Future studies are warranted to confirm this finding, and to explore reasons for it. It might be that these lean depressive subjects were those with some pre-existing somatic disease such as preclinical cancer affecting to both physical and mental health, or those with melancholic depressive symptoms as decreased appetite and weight loss. However, our study population consisted of CVD risk subjects who were apparently healthy at baseline, and thus confounding by pre-existing disease was unlikely, although we have previously found that 10% of our study population developed CVD during 8-year follow-up [27]. In addition, smoking was most prevalent among subjects with normal weight. We weren’t able to restrict our analysis to never-smokers, but we adjusted the main results for current smoking. In a primary care population, limiting the analysis for a small and selective cluster of people is somewhat arbitrary. Nevertheless, it may be possible that smoking and some reverse causality contributes to the rather steep increase in all-cause mortality risk among depressive subjects with normal weight in our study (Fig. 3). However, their health-related quality of life was on average higher and their self-rated health more often better than among those with increased depressive symptoms in the other categories of BMI. In addition, there were few deaths among those with at least severe obesity. The study by Bhaskaran et al. using large data set from the UK Clinical Practice Research Datalink and the UK national death registry, reported that low BMI up to 24–27 kg/m² was associated with increased mortality risk for mental health conditions [3]. Taken together, these results may imply that increased depressive symptoms leading to poor appetite or self-care could increase mortality risk even after a long time period.

Consistent with previous research, also depressive subjects with overweight or obesity had a two-fold increased mortality risk compared to non-depressive subjects with BMI < 25 kg/m^2^ in our study [11, 25, 26]. In contrast, the mortality risk of depressive subjects with overweight and obesity was not increased compared to non-depressive subjects within similar BMI category. This might indicate that BMI is a stronger predictor of mortality when a subject is beyond normal weight, and further points out the exceptional prognostic risk depressive symptoms cause among subjects with normal weight.

We studied a representative sample of CVD risk subjects commonly treated in primary care with comprehensive baseline measurements and mortality data. The results were adjusted for several CVD risk factors. However, we were not able to restrict our analysis to never-smokers, and time-dependent variation in self-reported depressive symptoms and BMI was not controllable. We also used BMI < 25.0 kg/m^2^ as normal-weight category including also subjects with underweight (BMI < 18.5 kg/m^2^). However, there were only three such subjects. The cardiovascular risk subjects in our study were offered lifestyle counselling accompanied by preventive medication if indicated, which may be associated with the lower SMR compared to the mortality rate throughout Finland over the same period [28]. In the general population, prevalence on overweight and obesity are much higher than prevalence of depressive symptoms, and there is more robust evidence of the association of BMI and mortality than depressive symptoms and mortality. Also, among our apparently healthy study population there were only few subjects with high BDI scores. Hence, we find it justified to dichotomize subjects according to depressive symptoms and represent how the association between depressive symptoms and all-cause mortality varies with BMI.

In this study among middle-aged primary care population at risk for CVD, we found out that the effect of depressive symptoms on all-cause mortality varies with BMI. Subjects with BMI < 25.0 kg/m^2^ and increased depressive symptoms have the highest mortality rate. This indicates importance of accounting depressive symptoms in future studies assessing BMI and mortality.

## Supplementary information

Supplemental figures 1,2, table 1

## Data Availability

The datasets analyzed during the current study are available from the guarantor on reasonable request.
