# Supplementary material for: Depressive symptoms and mortality – effect variation by body mass index: a prospective study in a primary care population
Source: Int J Obes (Lond). 2023 Mar 28;47(6):512–9. doi: 10.1038/s41366-023-01296-3 (PMC10212761; doi:10.1038/s41366-023-01296-3)
Supplement: Supplementary file 1 — Supplemental figures 1,2, table 1 [file 41366_2023_1296_MOESM1_ESM.pdf]

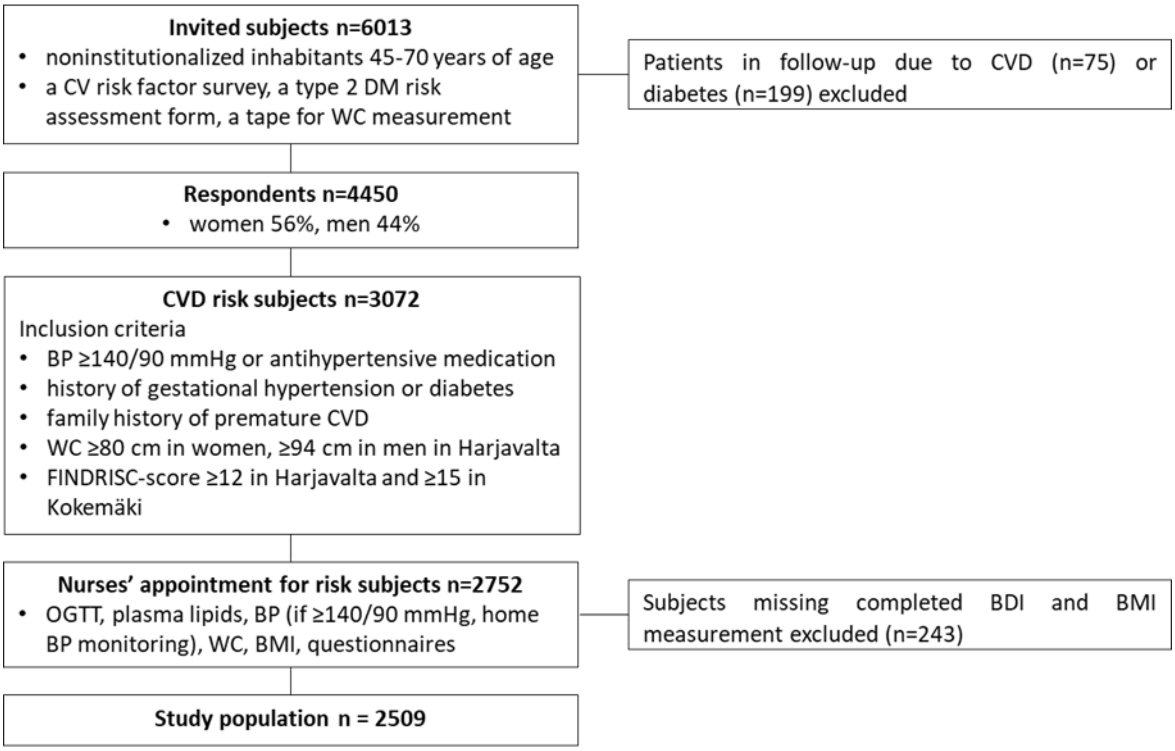


**Supplementary figure 1**. Flowchart of the study design. BP, blood pressure; BDI, Beck's Depression Inventory; BMI, body mass index; CVD, cardiovascular disease; FINDRISC, Finnish Diabetes Risk Score; OGTT, oral glucose tolerance test; SCORE, Systematic Coronary Risk Evaluation; WC, waist circumference.

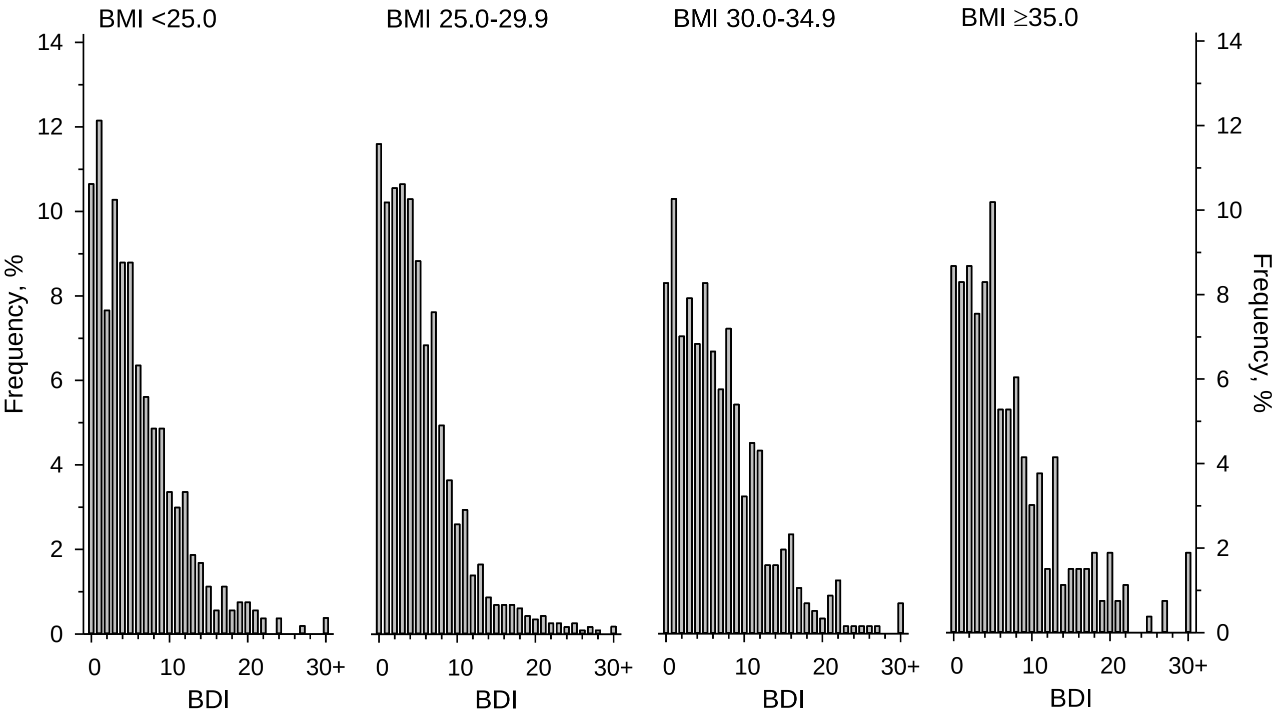


**Supplementary figure 2.** Distribution of Beck's Depression Inventory (BDI) score according to body mass index (BMI).

**Supplementary table 1**. Causes of death according to depressive symptoms and body mass index (BMI).

| **Causes of death**, n (%) | Body Mass Index category, kg/m$^2$ | | | | | | | |
|---|---|---|---|---|---|---|---|---|
| | <25.0 | | 25.0-29.9 | | 30.0-34.9 | | ≥35.0 | |
| | BDI<10 N=25 | BDI≥10 N=22 | BDI<10 n=112 | BDI≥10 n=23 | BDI<10 n = 44 | BDI≥10 n=23 | BDI<10 n=28 | BDI≥10 n=12 |
| Malignant neoplasms | 9 (36) | 10 (45) | 43 (38) | 11 (48) | 20 (45) | 13 (56) | 12 (43) | 5 (42) |
| Nervous system | 1 (4) | 3 (14) | 11 (10) | 1 (4) | 3 (7) | 0 (0) | 0 (0) | 0 (0) |
| Circulatory system | 6 (24) | 6 (27) | 36 (32) | 8 (35) | 12 (27) | 4 (17) | 7 (25) | 4 (33) |
| Digestive system | 0 (0) | 1 (5) | 2 (2) | 2 (9) | 2 (5) | 2 (9) | 4 (12) | 1 (8) |
| External cause | 3 (12) | 1 (5) | 10 (9) | 1 (4) | 5 (11) | 3 (13) | 3 (11) | 1 (8) |
| Other | 6 (24) | 1 (5) | 10 (9) | 0 (0) | 2 (5) | 1 (4) | 2 (7) | 1 (8) |

Beck´s Depression Inventory (BDI) <10, not increased depressive symptoms; BDI ≥10, increased depressive symptoms.
